# Integrating Germline and Somatic Mutation Information for the Discovery of Biomarkers in Triple-Negative Breast Cancer

**DOI:** 10.3390/ijerph16061055

**Published:** 2019-03-23

**Authors:** Jiande Wu, Tarun Karthik Kumar Mamidi, Lu Zhang, Chindo Hicks

**Affiliations:** 1 Department of Genetics and the Bioinformatics and Genomics Program, Louisiana State University Health Sciences Center, School of Medicine, 533 Bolivar Street, New Orleans, LA 70112, USA; jwu2@lsuhsc.edu (J.W.); tmamid@lsuhsc.edu (T.K.K.M.); 2 Louisiana Tumor Registry, Louisiana State University Health Sciences Center, School of Public Health, 2020 Gravier Street, New Orleans, LA 70112, USA; lzhan1@lsuhsc.edu

**Keywords:** germline somatic mutation, interactions cooperation, triple-negative breast cancer

## Abstract

Recent advances in high-throughput genotyping and the recent surge of next generation sequencing of the cancer genomes have enabled discovery of germline mutations associated with an increased risk of developing breast cancer and acquired somatic mutations driving the disease. Emerging evidence indicates that germline mutations may interact with somatic mutations to drive carcinogenesis. However, the possible oncogenic interactions and cooperation between germline and somatic alterations in triple-negative breast cancer (TNBC) have not been characterized. The objective of this study was to investigate the possible oncogenic interactions and cooperation between genes containing germline and somatic mutations in TNBC. Our working hypothesis was that genes containing germline mutations associated with an increased risk developing breast cancer also harbor somatic mutations acquired during tumorigenesis, and that these genes are functionally related. We further hypothesized that TNBC originates from a complex interplay among and between genes containing germline and somatic mutations, and that these complex array of interacting genetic factors affect entire molecular networks and biological pathways which in turn drive the disease. We tested this hypothesis by integrating germline mutation information from genome-wide association studies (GWAS) with somatic mutation information on TNBC from The Cancer Genome Atlas (TCGA) using gene expression data from 110 patients with TNBC and 113 controls. We discovered a signature of 237 functionally related genes containing both germline and somatic mutations. We discovered molecular networks and biological pathways enriched for germline and somatic mutations. The top pathways included the hereditary breast cancer and role of *BRCA1* in DNA damage response signaling pathways. In conclusion, this is the first large-scale and comprehensive analysis delineating possible oncogenic interactions and cooperation among and between genes containing germline and somatic mutations in TNBC. Genetic and somatic mutations, along with the genes discovered in this study, will require experimental functional validation in different ethnic populations. Functionally validated genetic and somatic variants will have important implications for the development of novel precision prevention strategies and discovery of prognostic markers in TNBC.

## 1. Introduction

Triple-negative breast cancer (TNBC) rep­resents a diverse group of cancers that are characterized by lack of expression of the estrogen receptor (ER), progesterone receptor (PR), and human epidermal growth factor receptor 2 (HER2) amplification [1,2,3]. TNBC is a heterogeneous disease representing 17–20% of all breast cancers diagnosed in the general US population [1,2,3]. Patients with TNBC have a significantly increased risk of relapse and shorter survival time than patients affected by other molecular subtypes of breast cancer [1,2,3]. Unlike ER/PR+ and HER2+ breast cancers which are responsive to targeted therapy, there is no effective targeted therapy for TNBC [4]. Cytotoxic chemotherapy remains the only effective therapeutic modality. Therefore, there is an urgent need for the discovery of molecular markers for the development of precision prevention strategies and novel therapeutics. 

Well-established risk factors for TNBC include age, ethnicity, family history, and genetics. Patients with TNBC usually show an early onset of the disease, as well as a positive family history of breast cancer, suggesting that TNBC may be closely associated with a hereditary disease cause. Germline mutations in the *BRCA1* and *BRCA2* genes have been associated with up to 15% of TNBC [5]. Importantly, TNBC accounts for 70% of breast tumors arising in *BRCA1* mutation carriers and 16–23% of breast tumors in *BRCA2* carriers [5]. However, it is not clear whether germline mutations in other breast cancer susceptibility genes also predispose to TNBC, or whether the *BRCA1* and *BRCA2* genes interact and cooperate with other genetic susceptibility genes to drive TNBC. 

Over the last decade, transcription profiling using microarray technology has enabled molecular classification of the subtypes of TNBC and discovery of clinically actionable biomarkers [6,7]. However, the causal association between gene expression and TNBC remains to be determined. Emerging evidence indicates that germline mutations may interact with somatic alterations to drive carcinogenesis [8]. The association of genetic susceptibility risk variants with somatic mutation burden in breast cancer in general has been reported [9]. However, the possible oncogenic interactions and cooperation between germline and somatic alterations in TNBC remains largely unknown. 

Advances in high-throughput genotyping and reduction in genotyping costs have enabled the discovery of germline mutations, primarily single-nucleotide polymorphisms (SNPs) associated with an increased risk of developing breast cancer using genome-wide association studies (GWAS) [10,11]. Discoveries from these studies are providing valuable insights about the genetic susceptibility landscape of breast cancer. The recent surge of next generation sequencing of the cancer genomes has led to an expanded molecular classification of types and subtypes of breast cancer, and the discovery of somatic driver mutations acquired during tumorigenesis [12]. Large multicenter projects, such as The Cancer Genome Atlas (TCGA) [13] and the International Cancer Genome Consortium (ICGC) [14], have performed a series of detailed analyses of the somatic alterations affecting tumor genomes in breast cancer and other cancers. Discoveries from these large-scale studies are providing valuable information about the genomic landscape of breast cancer. However, to date, somatic mutation information has not been maximally leveraged and integrated with information on germline genetic susceptibility variants to infer the possible oncogenic interactions and cooperation between germline and somatic variation in TNBC. This limited progress must be balanced against the recognition that in the past, germline mutations contained within the heritable genome, and somatic mutations, acquired de novo by cancer cells, have historically been considered as separate endeavors in cancer research. 

The objective of this exploratory study was to investigate the possible oncogenic interactions and cooperation between genes containing germline mutations and genes containing somatic mutations or both in TNBC and to identify the molecular networks and biological pathways through which they cooperate. Our working hypotheses included (1) genes containing germline mutations also harbor somatic mutations and are functionally related and (2) TNBC originates from a complex interplay between germline and somatic-mutated genes, and that these complex array of interacting genetic factors affect entire molecular networks and biological pathways, which in turn drive the disease. To address these hypotheses, we combined germline mutation information derived from GWAS with somatic mutation information derived from tumor samples, using gene expression data on TNBC and control samples from TCGA. For the purposes of clarity, in this study, we defined genetic variants or single-nucleotide polymorphisms (SNPs) associated with an increased risk of developing breast cancer as germline mutations and the genes containing these mutations as the units of association. Likewise, we have defined mutations derived from the tumor genomes as somatic mutations and used the genes containing these mutations in the analysis. Thus, rather than considering actions of individual mutations, our analysis has taken a holistic approach by using mutated genes as the variables and gene expression data as the intermediated phenotype to understand the broader biological context in which germline and somatic mutations operate and cooperate to drive TNBC.

## 2. Materials and Methods

### 2.1. Germline Mutations and Associated Genes

We have previously published a comprehensive catalog of genetic variants primarily single-nucleotide polymorphisms (herein referred to as germline mutations) associated with an increased risk of developing breast cancer [10]. For this study, we used an updated version of this catalog and supplemented it with information from the GWAS catalog which is continuously updated [10,15] to accommodate the most recent publications on GWAS [10,15]. The methods used to collect genetic variants and genes from GWAS have been described in our earlier publication [10], which followed the guidelines proposed by the Human Genome Epidemiology Network for systematic review of genetic associations [16,17,18,19,20]. 

In this study, we reviewed 250 published reports on GWAS to identify genetic variants and genes associated with increased risk of breast cancer. The reports were screened by title, abstract, and full-text review to identify the studies meeting the following eligibility criteria; (1) using a case-control study design with unrelated individuals; (2) publications must have been of full length and published in peer-reviewed journals or online in English language before October 2018; (3) breast cancer must have been diagnosed by histological examination; (4) the sample sizes must be more than 500 for the cases and more than 500 for the controls to reduce sampling errors; (5) the study must have provided sufficient information such that genotype frequencies for both breast cancer and controls can be discerned without ambiguity; and (6) the studies must have used the appropriate and recommended statistical methods to infer the associations by taking into account the covariates and accounting for population structure and genetic background [16,17]. 

We manually extracted the information from the 230 studies meeting our eligibility criteria and the accompanying websites containing Supplementary Data. The extracted information included SNP identification number (rs-ID); evidence of association as determined by the GWAS *p*-value; a composite of strong (*p ≤* 10^−7^), moderate (P = 10^−5^–10^−6^), and weak (P = 10^−2^–10^−4^) association; gene name; and associated chromosome positions to which the genes map as determined by the dbSNP database [21] and the Human Genome Nomenclature database [22]. The combined data set included 632 genes containing genetic variants associated with an increased risk of developing breast cancer derived from GWAS reports. A complete list of genetic variants and genes along with sources or published reports from which they were derived is presented in Table SA provided as Supplementary Data to this report. 

### 2.2. Somatic Mutation Information and Gene Expression Data

We downloaded somatic mutation, gene names and clinical information from TCGA via the Genomics Data Commons https://gdc.cancer.gov/ [23]. Using patient IDs, gene symbols, and clinical information, we created a catalog of somatic mutations and mutated genes on 110 TNBC patients. We analyzed the somatic mutation events per gene to identify genes which are highly mutated. Additionally, we analyzed the frequency of mutations across patients to assess genetic heterogeneity and percentage of patients carrying particular mutation in each mutated gene. The gene was considered highly mutated if it had >3 mutation events. The mutation frequencies were classified as high if mutated in >10% of patients or intermediate if mutated in ≤10% of patients. From this analysis, we created a comprehensive catalog of somatic mutations and mutated genes used in the analysis. 

Gene expression data on the same patient population generated using RNA-seq was downloaded from TCGA via the Genomics Data Commons (GDC) data transfer tool along with clinical information at https://gdc.cancer.gov/ [23]. The gene expression data set included 110 patients diagnosed with TNBC and 113 normal samples. The data matrix was filtered to remove rows with missing data, such that each row had at least ≥30% data using CPM (counts per million) filter (>0.5) implemented in R. The resulting data set was normalized by the trimmed mean of M-values (TMM) [24] normalization method and then transformed by Voom [24], using the “LIMMA” package implemented in R [24]. The normalized data contained 36,451 probes and was used in the analysis. The probe IDs and gene names were matched for interpretation using the Ensemble database, a database used for gene annotation of sequencing experiments on sequencing technology platforms used by TCGA.

### 2.3. Data Analysis

**Figure 1:**
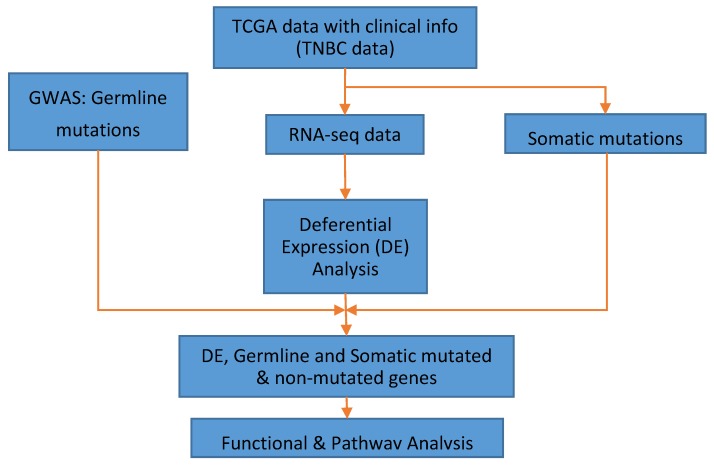
Overall project design showing sources of data and integrative genomic and functional analysis workflow for combining germline and somatic-mutated genes and nonmutated genes. DE indicates differential expression. GWAS denotes genome-wide association studies, TCGA represents The Cancer Genome Atlas, and TNBC represents triple-negative breast cancer.

The overall project design and data analysis workflow is presented in Figure 1. We used three data sets: GWAS data containing germline mutations and associated genes, somatic mutations and associated genes and whole genome transcriptome data derived from RNA-Seq as described in the preceding sections and shown in Figure 1. As a first step, we performed whole genome analysis comparing gene expression levels between patients diagnosed with TNBC and matched control samples using Bioconductor package “LIMMA” [24] to identify significantly differentially expressed genes between tumors and control samples. This unbiased approach was carried out to discover germline and somatic-mutated genes as well as nonmutated genes associated with TNBC. We computed both the observed p-values and adjusted *p*-values to correct for multiple hypothesis testing (i.e., false discovery rate, (FDR)) [25]. The genes were ranked based on adjusted *p*-values. In addition, we evaluated the number of somatic and germline mutation events per gene and across samples as described in the preceding section.

We performed hierarchical clustering using “Morpheus” [26] to determine whether the genes significantly associated with TNBC are coregulated and have similar patterns of expression profiles. First, we performed hierarchical clustering on significantly differentially expressed genes containing both germline and somatic mutations distinguishing patients with TNBC from control samples. Secondly, we performed clustering combining genes containing germline mutations only with genes containing somatic mutations only. In each clustering step we used the Pearson correlation coefficient as a measure of distance between pairs of genes and the complete linkage method for clustering. 

We performed network and pathways analysis using Ingenuity Pathway Analysis (IPA) software to identify molecular networks and biological pathways enriched for germline and somatic mutations [27]. Using IPA, the most highly significantly differentially expressed genes (containing both germline and somatic mutations) distinguishing patients with TNBC from control samples were mapped onto networks and canonical pathways. We performed additional analysis combining genes containing germline mutations only with genes containing somatic mutations only. In each analysis, the probability scores and the log *p*-values were calculated to assess the likelihood and reliability of correctly assigning the mutated genes to the correct molecular networks and biological pathways. A false discovery rate was used to correct for multiple hypothesis testing in pathway analysis. The predicted molecular networks and biological pathways were ranked based on z-scores and log *p*-values, respectively. We performed Gene Ontology (GO) analysis [28] as implemented in IPA, to gain insights about the molecular functions, biological processes, and cellular components in which the genes containing germline and somatic mutations are involved.

## 3. Results

### 3.1. Significant Differentially Expressed Mutated and Nonmutated Gene Signatures

**Figure 2:**
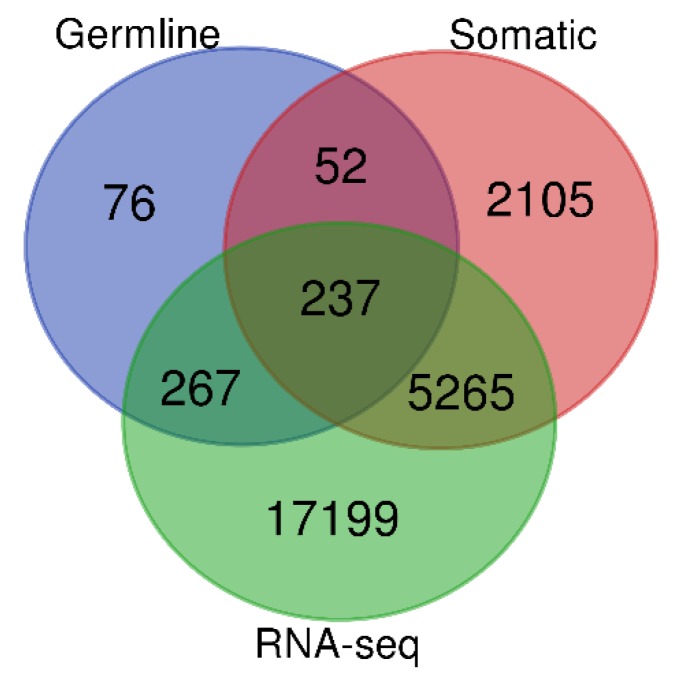
Distribution of somatic and germline-mutated and nonmutated genes significantly differentially (and not differentially) expressed between patients with TNBC and control samples. The blue circle represents genes containing germline mutations, green circle represents all significantly differentially expressed genes from RNA-Seq, and the pink circle represents all somatic-mutated genes.

To address the hypothesis that germline, somatic-mutated and nonmutated genes are significantly differentially expressed between patients with TNBC and control samples, we performed whole genome analysis comparing gene expression levels between patients with TNBC and control samples. The results showing the distribution of the number of genes in each gene signature are shown in the Venn diagram in Figure 2. Using an adjusted *p*-value (*p* < 0.05), the analysis revealed a signature of 5502 significantly differentially expressed somatic-mutated genes and 17,466 significantly differentially expressed genes without somatic mutations distinguishing patients with TNBC from control samples. Table 1 presents the top 34 highly significantly differentially expressed and highly somatic-mutated (>5 mutation events) genes. Also presented in the Table 1 are chromosome positions indicating the location of the mutated genes, the number of somatic mutation events for individual genes, and the adjusted *p*-values indicative of significance in the level of expression. A complete list of significantly differentially expressed somatic-mutated and nonmutated genes distinguishing patients with TNBC from controls are presented in Table S1A (for mutated) and Table S1B (for nonmutated genes).

### 3.2. Germline and Somatic Mutation Gene Signatures

To discover and characterize the genes containing both germline and somatic mutations significantly differentially expressed between TNBC and controls, we evaluated all the 632 genes containing germline mutations. Out of the 632 genes evaluated, 289 genes contained both germline and somatic mutations confirming our hypothesis that genes containing germline mutations also harbor somatic mutations (Figure 2). The remaining 343 contained only germline mutations (Figure 2). Among the genes containing germline and somatic mutations, 237 genes were significantly (*p* < 0.05) differentially expressed, whereas 52 genes were not significantly differentially expressed (Figure 2). Among the genes containing germline mutations only, 267 genes were significantly differentially expressed, whereas the remaining 76 genes were not significantly associated with TNBC (Figure 2). A complete list of all the 632 genes containing germline mutations along with their estimates of adjusted gene expression *p*-values are presented in Table S2 provided as Supplementary Data.

Further evaluation focusing on somatic-mutated genes only revealed a signature of 5265 significantly differentially expressed genes (Figure 2) and 2105 genes not significantly differentially expressed. Table 2 presents a signature of 29 genes containing both germline and somatic mutations (somatic mutations events ≥3) which were highly significantly differentially expressed at >2-fold change. Also presented in the table are the germline mutations, GWAS association *p*-value, adjusted *p*-value from gene expression and the number of somatic mutation events per gene. 

### 3.3. Patterns of Expression Profiles for Genes Containing Germline and Somatic Mutations

**Figure 3:**
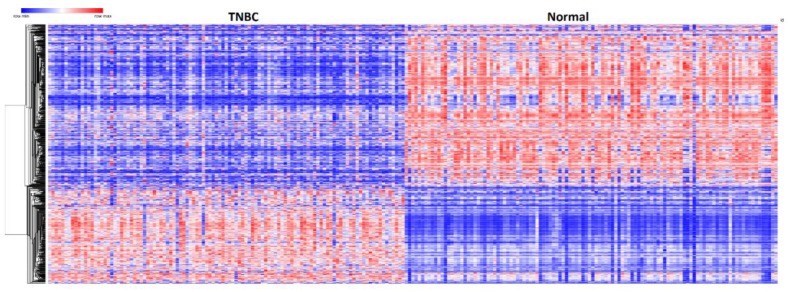
Patterns of gene expression profiles for the 237 up and downregulated genes containing both germline and somatic mutations found to be significantly (*p* < 0.05) differentially expressed between patients with TNBC and control samples. Red color represents upregulation; blue color represents downregulation.

To investigate whether genes containing germline and somatic mutations are coregulated and have similar patterns of expression profiles, we performed hierarchical clustering using the 237 genes containing both germline and somatic mutations associated with TNBC. Figure 3 shows the patterns of expression profiles for the 237 up- and downregulated genes containing both germline and somatic mutations significantly associated with TNBC. A complete list including names of the 237 genes containing germline and somatic mutations significantly associated with TNBC used to generate Figure 3 is provided in Table S3 provided as Supplementary Data. The analysis revealed that genes containing both germline and somatic mutations are coexpressed and have similar patterns of expression profiles. Interestingly, genes containing weak to moderate GWAS associations were coregulated with genes containing genetic variants with strong associations. There was significant variation in the patterns of gene expression profiles. 

The variability in patterns of expression profiles among the genes containing germline and somatic mutations (Figure 3) can partially be explained by the diversity of populations and clinical phenotypes from which they were derived. In addition, TNBC is inherently a heterogeneous disease entity comprising of many subtypes with varying patterns of expression profiles across subtypes [3]. Overall, the results of hierarchical clustering suggest that genes containing both germline and somatic mutations cooperate through coregulation and functional relationships.

Because gene regulation involves *cis* and *trans* regulation, restricting analysis to only genes containing both germline and somatic mutations could miss important information. Genes containing somatic mutations only may be regulated by genes containing germline mutations only and vice versa. To address this knowledge gap, we performed addition hierarchical clustering using a set of 154 genes. This set included the 99 genes containing somatic mutations only with >2 somatic mutation events per gene, and were significantly associated with TNBC with a fold change of (Log2(FC) ≥ 2) and a set of 55 significantly differentially expressed genes containing germline mutations only selected using the same criteria and threshold. Our working hypothesis was that highly somatic-mutated genes are likely coregulated and have similar patterns of gene expression profiles with genes containing germline mutations only strongly associated with TNBC. 

**Figure 4:**
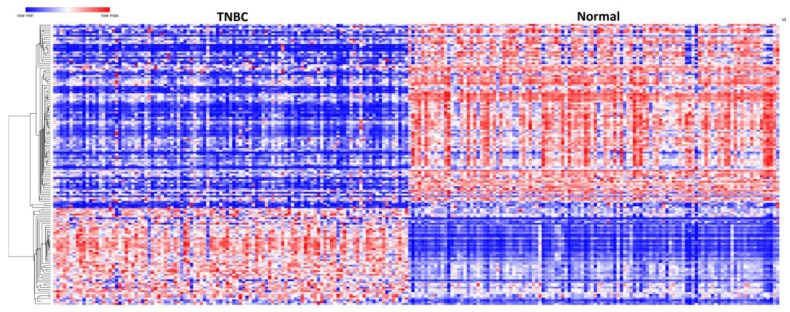
Patterns of gene expression profiles for the 154 gene signatures derived from the 55 genes containing germline mutations only and 99 genes containing somatic mutations only. The red color represents upregulation and blue color represents downregulation.

The results showing patterns of expression profiles for the 154 gene signature are presented in Figure 4. Hierarchical clustering revealed that genes containing germline mutations only and genes containing somatic mutations only are coregulated and have similar patterns of expression profiles confirming our hypothesis (Figure 4). Genes containing germline mutations with strong association were coregulated with genes containing germline mutations with strong associations and with genes containing somatic mutations only. A complete list of 99 significantly differentially expressed somatic-mutated genes only and the 55 germline-mutated genes significantly associated with TNBC are presented in Table S4A (for somatic-mutated genes only) and Table S4B (for germline-mutated genes only), provided as Supplementary Data to this report. 

Having established that genes containing both germline and somatic mutations are coregulated, we performed additional analysis involving 366 genes (all the 267 genes containing germline mutations only and the 99 highly somatic-mutated genes) that were significantly associated with TNBC. The analysis revealed similarities in patterns of expression profiles (see Supplementary Figure S1). Further analysis combining germline muted, somatic-mutated and nonmutated genes also revealed similarities in patterns of gene expression profiles (see Supplementary Figure S2). This indicates that cooperation among the genes may not be limited to genes containing both germline and somatic mutations only but is also likely to involve other germline and somatic-mutated genes. The results of coexpression and similarity in patterns of gene expression among the germline-mutated, somatic-mutated, and nonmutated genes suggest that cooperation among these genes likely occurs through coregulation and functional relationships.

### 3.4. Molecular Networks and Biological Pathways

**Figure 5:**
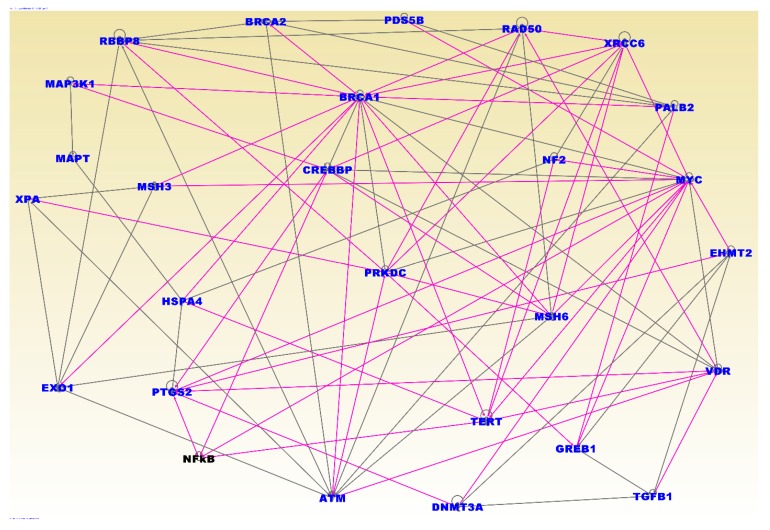
Network analysis showing interactions and functional relationships among the 237 genes containing both germline and somatic mutations. All genes in blue font contained both germline and somatic mutations and were significantly differentially expressed between patients with TNBC and controls. Many of these genes were also found to interact with the *NF-kB* complex (black font). The line colors indicate overlap in functional relationships among genes in different networks. Note that in the networks there are fewer genes than the input genes due to filtering that we imposed on the networks to remove spurious interactions and less significant networks.

To gain insights about the broader biological context in which germline and somatic mutations operate and to understand the potential mechanisms of cooperation we performed network and pathway analysis using the 237 genes containing both germline and somatic mutations that were significant associated with TNBC. Figure 5 presents the results of network and pathway analysis. The analysis revealed functionally related genes interacting in molecular networks and biological pathways enriched for germline and somatic mutations. Among the most significant networks included the networks containing genes predicted to be involved in cancer (Z-score = 48), cellular function and maintenance (Z-score = 43), DNA replication and repair (Z-score = 35), and cell cycle (Z-score 27). The discovered networks included the genes *BRCA1*, *BRCA2*, *ATM*, *CHEK1*, *CHEK2*, *BRIP1*, *NBN*, *PALB2*, *RAD51C*, *RA51D*, *FANCM*, *MRE11A*, *RAD50*, *ESR1*, *TOX3*, *ANKLE1*, *LGR6*, *MDM4*, *TERT*, *PEX14*, *ADAM29*, *EBF1*, *TCF7L2*, *PTHLH*, *NTN*, *MLK1*, *RAD51L1*, *TGFB1*, *LSP1*, *MAP3K1*, *CASP8*, and *TP53* containing germline mutations confirmed to be directly associated with TNBC (see references in Supplementary Table SA). This confirmed our hypothesis that cooperation between germline and somatic-mutated genes likely occurs through molecular networks. 

Pathway analysis produced biological pathways enriched for germline and somatic mutations. The most highly significant pathways included the hereditary breast cancer, role of *BRCA1* in DNA damage response, DNA double-strand break repair, estrogen-dependent breast cancer, FGF, EGF, molecular mechanisms of cancer, and the p53 signaling pathways. The top upstream regulators included the genes *PTTG1*, *SPP1*, and *TP53*, all of which have been directly associated with TNBC. Interestingly, although *TP53* was not highly differentially expressed, it contained both germline and somatic mutations and was the most highly somatic-mutated gene. This further confirmed our hypothesis that oncogenic interactions between germline and somatic-mutated genes likely occurs through biological pathways. 

One of the limitations of GWAS studies has been the missing variation; a phenomenon that refers to the small proportion of the phenotypic variation explained by genetic variants reported that far. Under this phenomenon, the limiting network and pathway analysis of genes containing both germline and somatic mutations only could miss important information. To address this issue, we performed additional network and pathways analysis on the 154 gene signature, derived from combining the 55 genes containing germline mutations only that were highly significantly associated with TNBC and the 99 genes containing somatic mutations only significantly associated with TNBC and have highest somatic mutation events. 

**Figure 6:**
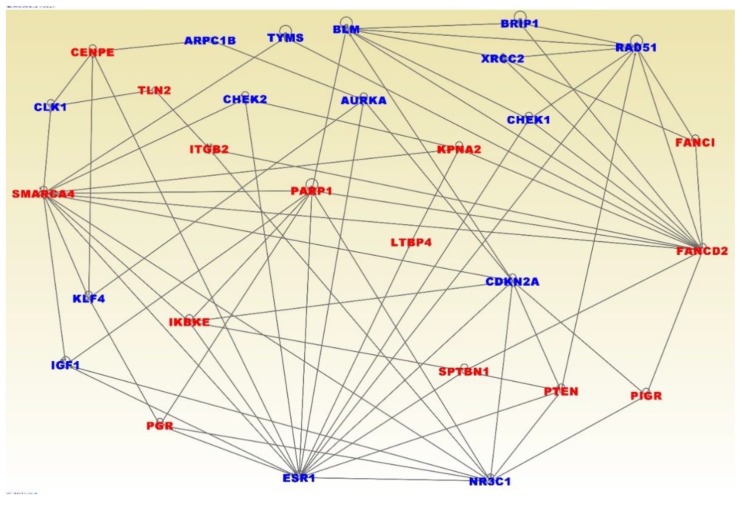
Network analysis showing interactions and functional relationships among and between the genes containing germline mutations only and genes containing somatic mutations only. Genes in blue contain germline mutations and genes in red fonts contain somatic mutations and were all significantly differentially expressed between patients with TNBC and controls.

Figure 6 presents the results of network analysis for the 154 gene signature. The analysis revealed interactions among and between the genes containing germline mutations only (in blue fonts) and genes containing somatic mutations only (in red fonts) (Figure 6). The most significant gene regulatory networks included genes predicted to be involved in cell cycle (Z-score = 45), cancer (Z-score = 30), DNA replication and repair (Z-score = 30), and cell death (Z-score 23). Interestingly, the discovered gene regulatory networks included the genes *BRCA1*, *BRCA2*, *ATM*, *CHEK1*, *CHEK2*, *BRIP1*, *NBN*, *PALB2*, *RAD51C*, *RA51D*, *FANCM*, *MRE11A*, *RAD50*, *ESR1*, *TOX3*, *ANKLE1*, *LGR6*, *MDM4*, *TERT*, *PEX14*, *ADAM29*, *EBF1*, *TCF7L2*, *PTHLH*, *NTN*, *MLK1*, *RAD51L1*, *TGFB1*, *LSP1*, *MAP3K1*, *CASP8*, and *TP53* containing germline mutations confirmed to be directly associated with TNBC (see Supplementary Table SA for references).

Pathway analysis produced biological pathways enriched for germline and somatic mutations. The most highly significant pathways included the hereditary breast cancer, role of *BRCA1* in damage response, *ATM* signaling, hereditary breast cancer, DNA damage checkpoint regulation, *P53* signaling, molecular mechanisms of cancer, and *PTEN, STAT3*, and *ERS* signaling pathways. The top upstream regulators included the genes *ERBB2, SMARCA4, MAPK*, and the *ESR1*. Overall, the investigation revealed oncogenic interactions and cooperation among genes containing both germline and somatic mutations and also with other genes. 

## 4. Discussion

For genetic research on TNBC, analysis of germline and somatic mutations has largely been conducted as separate endeavors. Here we report the possible oncogenic interactions and cooperation among genes containing germline and somatic mutations. We used several analytical approaches to map the germline–somatic mutation interaction landscape. We discovered that genes containing germline mutations also harbor somatic mutations. Most notably, the investigation revealed that oncogenic interactions and cooperation among germline and somatic-mutated genes occurs in least three ways through (1) coregulation and (2) gene regulatory networks and biological pathways. The novel aspect of the study is that it provides valuable insights about the broader biological context in which oncogenic interactions and cooperation between germline and somatic alterations occur to drive TNBC. 

The discovery of biological pathways enriched for germline and somatic mutations including the role of *BRCA1* in DNA repair, ATM, hereditary breast cancer, and DNA damage checkpoint signaling pathways is of particular interest. Because these pathways are involved in the DNA repair machinery and maintenance of the genome integrity, they could be used as therapeutic targets. Additionally, germline mutational status may serve as a robust biomarker for predicting response to therapy, particularly with respect to compounds challenging the DNA repair machinery. 

Among the genes containing both germline and somatic mutations included the genes *ATM*, *BRCA1*, *BRCA2*, *PTEN*, *TP53*, *NBN*, *BRIP1*, *FANCM*, *PALB1*, *CHEK1*, *CHEK2*, and *RAD50.* These genes contain genetic variants reported to be directly associated with TNBC [29]. The discovery of germline and somatic-mutated genes interacting with *BRCA1* and *BRCA2* genes is of particular interest, because over 70% of TNBC patients tend to have *BRCA1* mutations [5,29]. Moreover, mutations in *BRCA1* and *BRCA2* genes affect both hereditary and sporadic breast cancers [30,31,32]. The clinical significance of these findings is that *BRCA1* and *BRCA2* genes could be used together with other mutated genes to create refined gene panels to be used for screening patients and monitoring disease progression [33]. For example, a recent multigene hereditary cancer panel, including the genes *BARD1*, *BRCA1*, *BRCA2*, *PALB2*, and *RAD51D*, containing germline and somatic mutations found in this study, revealed that pathogenic germline variants in these genes are associated with high risk (odds ratio > 5.0) of TNBC, and greater than 20% lifetime risk for overall breast cancer among Caucasians [34]. Pathogenic variants in genes *BRIP1*, *RAD51C*, and *TP53* also containing germline and somatic mutations in this study have been associated with moderate risk (odds ratio > 2) of TNBC [34]. 

In addition to multigene panels, germline mutations could be used for the development and refinement of novel precision prevention strategies such as polygenic risk scores (PRSs) optimized for prediction of estrogen receptor (ER)-specific disease [35]. Stratification of women according to their risk and type of breast cancer based on polygenic risk scores (PRSs) could improve screening and precision prevention. Although we did not compute the polygenic risk scores in this investigation, the use of germline mutations including those reported in this study to compute risk score in breast cancer has been reported [35]. 

In this investigation, we did not investigate the impact of germline mutations on the somatic genome. However, our previous study showed that germline mutations associated with an increased risk of developing breast cancer, could disrupt *cis* and *trans* regulatory elements such as enhancers, splice sites and binding sites [36]. Moreover, a pan-cancer analysis of enhancer expression across nearly 9000 patient samples of 33 cancer types from the TCGA revealed that enhancers are key regulators of therapeutic targets [37]. 

It is worth noting that the link between germline and somatic alterations in breast carcinogenesis in general has been explored [38]. The novel aspect of our study is that it focused on exploring the possible oncogenic interactions and cooperation between germline and somatic-mutated genes using network and pathway based approaches in TNBC. Although we did not investigate the mechanism by which germline mutations can potentiate the somatic revolution, recent studies involving interactions between germline and somatic mutations in cancer have revealed that genetic background can influence the somatic mutation revolution in at least two ways: (1) in determining the site of tumorigenesis and (2) by modifying the likelihood of acquiring somatic mutations in specific coregulated and functionally related genes [8,39,40], which is consistent with the results in this study. This study adds another dimension by revealing that germline mutations may influence the somatic revolution through gene regulatory networks and biological pathways. 

One of the clinical applications of molecular markers in breast cancer is assessing disease prognosis. The PAM50 gene signature has gained prominence in clinical applications as a prognostic gene signature in breast cancer [41,42]. The prognostic value of PAM50 intrinsic gene signature has been shown to be predictive of risk of recurrence, a common feature in TNBC and benefit of chemotherapy, which is the only effective therapeutic modality for TNBC [41,42]. To investigate whether the germline and somatic-mutated genes discovered in this study have prognostics value, we evaluated them against the PAM50 gene signature. Evaluation of the genes containing both germline and somatic mutations significantly associated with TNBC revealed eight genes: *CCNE1, CEP55, EGFR, ESR1, EXO1, FGFR4, MAPT*, and *MYC* reported in PAM50. In addition, the evaluation of genes containing germline mutations only, significantly associated with TNBC, revealed five genes *BCL2, ESR1, PGR, PHGDH*, and *TYMS* reported in PAM50. Importantly, these genes were found to be coregulated and interacting with other genes in molecular networks and biological pathways. These findings suggest that genes containing germline and somatic mutations may have prognostic value.

Machiela et al. observed limited evidence for cancer susceptibility regions as preferential targets for somatic mutations [43]. The main difference between that report and this study is that in this investigation, we used network and pathway- based approach not undertaken in the previous report [43]. More importantly, Machiela et al. [43] concluded that despite limited evidence that cancer susceptibility regions are preferential targets for somatic mutations, interactions may occur through complex gene regulatory networks and biological pathways, which in agreement with findings from this study. 

Limitations of the study: We are mindful of the limitations of using GWAS information as the source of germline mutations. Thus far, translating GWAS discoveries into clinical practice has been limited, in part because germline risk variants identified to date have small effect sizes [44]. Our investigation suggests that this limitation could potentially be overcome by using an integrative genomics approach. GWAS have not been breast cancer type-specific. Thus, further genotyping studies confirming and validating germline mutations in TNBC will provide more insights. Importantly, GWAS have been almost exclusively conducted on women of European ancestry. Lack of diversity in genomics data and databases is a potential barrier to translating discoveries from GWAS into clinical practice to guide therapeutic decisions, realization of precision medicine, and elimination of health disparities [45]. Therefore, there is need for further studies involving diverse populations. However, despite these limitations, all of which are beyond the scope of this investigation, the study shows the power of using an integrative genomics approach to map the possible oncogenic interactions and cooperation between germline and somatic mutations and to understand the broader biological context in which they operate in TNBC. Although our study focused on TNBC, the approach could be applied to other types of cancer and common human diseases in which both germline and somatic mutations play a role.

## 5. Conclusions

In conclusion, this is the first large-scale and comprehensive analysis delineating the possible oncogenic interactions and cooperation among and between genes containing germline and somatic mutations in TNBC. Integrating germline and somatic mutation information holds the promise of defining the molecular networks and biological pathways driving TNBC. Genetic and somatic mutations as well as the genes discovered in this study will require experimental functional validation in different ethnic populations. Functionally validated genetic and somatic variants will have important clinical implications for the discovery of molecular markers and therapeutic targets and development of novel precision prevention strategies in TNBC. 

## Figures and Tables

**Table 1:** List of the 34 gene signature containing the most highly somatic-mutated genes (>5 mutation events per gene) that were significantly differentially expressed between TNBC tumors and controls.

Gene Symbol	Chromosome Position	Expression *p*-Value	Number of Mutation Events
TTN *	2q31.2	0.012712726	27
***MUC16***	19p13.2	9.79 × 10^6^	11
***OBSCN***	1q42.13	0.001208653	10
***SPTA1***	1q23.1	4.58 × 10^−5^	9
SYNE1 *	6q25.2	1.11 × 10^−44^	9
DNAH17 *	17q25.3	4.74 × 10^−5^	8
***DST***	6p12.1	1.88 × 10^−50^	8
***MUC5B***	11p15.5	8.87 × 10^−21^	8
PIK3CA *	3q26.32	2.59 × 10^−9^	8
AHCTF1 *	1q44	2.54 × 10^−9^	7
***ASPM***	1q31.3	3.27 × 10^−68^	7
***CREBBP***	16p13.3	3.83 × 10^−6^	7
***CSMD2***	1p35.1	1.38 × 10^−12^	7
***FLG***	1q21.3	0.018204904	7
KMT2D *	12q13.12	9.52 × 10^−7^	7
***PLEC***	8q24.3	0.000153621	7
***SMG1***	16p12.3	0.040114062	7
***USP34***	2p15	0.000850126	7
***AHNAK***	11q12.3	2.29 × 10^−61^	6
***ARID1B***	6q25.3	7.25 × 10^−5^	6
***CACNA1B***	9q34.3	9.79 × 10^−8^	6
***CENPE***	4q24	2.29 × 10^−60^	6
***COL18A1***	21q22.3	0.007574248	6
***F5***	1q24.2	6.47 × 10^−8^	6
IGSF10 *	3q25.1	2.63 × 10^−38^	6
***KIF26B***	1q44	9.80 × 10^−26^	6
***LAMA3***	18q11.2	3.55 × 10^−41^	6
LRP1 *	12q13.3	5.49 × 10^−33^	6
***LYST***	1q42.3	1.39 × 10^−21^	6
***MFI2***	3p29	5.09 × 10^−13^	6
***SAGE1***	Xq26.3	0.045910304	6
***SPTBN1***	2p16.2	2.23 × 10^−34^	6
***STAB1***	3p21.1	2.20 × 10^−6^	6
***ZNF512B***	20q13.33	2.73 × 10^−8^	6

* Indicates that the gene contains germline mutations that have been directly associated with TNBC.

**Table 2:** The 29 genes signature of the most highly significantly differentially expressed genes containing both germline and somatic mutations.

Genes	Chromosome Position	Genetic Variant	GWAS *p*-Value	Expression *p*-Value	Mutation Event
***CREBBP***	16p13.3	rs12920416	8.00 × 10^−7^	3.83 × 10^−6^	7
ARID1B *	6q25.3	rs140842923	3.00 × 10^−6^	7.25 × 10^−5^	6
BRCA1 *	17q21.31	rs6558174	3.00 × 10^−6^	3.95 × 10^−7^	5
ERBB4 *	2q34	rs13393577	9.00 × 10^−14^	9.03 × 10^−41^	5
***FHOD3***	18q12.2	rs9956546	2.90 × 10^−6^	1.62 × 10^−19^	5
***TNRC6B***	22q13.1	rs12483853	1.00 × 10^−18^	9.59 × 10^−6^	5
***ARHGAP24***	4q21.23-q21.3	rs71599425	6.00 × 10^−6^	4.05 × 10^−44^	4
ARHGAP5 *	14q12	rs140783387	3.00 × 10^−7^	6.22 × 10^−15^	4
***CNTNAP2***	7q35-q36.1	rs72826962	5.00 × 10^−9^	2.35 × 10^−5^	4
***DMD***	Xp21.2-p21.1	rs145455135	9.00 × 10^−6^	2.42 × 10^−40^	4
EFR3B *	2p23.3	rs1971136	5.009	0.000451	4
***MSH3***	5q14.1	rs1863333	0.0056	2.45 × 10^−30^	4
***MYO10***	5p15.1	rs2562343	0.0092	1.56 × 10^−25^	4
***MYT1***	20q13.33	rs6062356	3.00 × 10^−6^	0.00559	4
***RELN***	7q22.1	rs17157903	0.0006	8.36E-21	4
***SPAG17***	1p12	rs1962373	1.00 × 10^−6^	7.67 × 10^−5^	4
***TRIM46***	1q22	rs4971059	5.00 × 10^−11^	1.14 × 10^−17^	4
***ZFPM2***	8q23	rs12546444	8.00 × 10^−11^	3.46 × 10^−20^	4
***ADCY9***	16p13.3	rs56278937	1.00 × 10^−6^	1.31 × 10^−25^	3
AKAP9 *	7q21.2	rs10644111	3.00 × 10^−11^	2.70 × 10^−6^	3
ASH1L *	1q22	rs10796944	7.00 × 10^−10^	5.39 × 10^−6^	3
***ASXL2***	2p23.3	rs144079028	9.00 × 10^−6^	0.000116	3
ATM *	11q22.3	rs1801516	0.0002	1.35 × 10^−8^	3
***ATXN1***	6p22.3	rs3819405	2.00 × 10^−8^	3.92 × 10^−6^	3
BAHCC1 *	17q25.3	rs8074440	3.00 × 10^−6^	0.02797	3
***CASZ1***	1p36.22	rs199867187	1.00 × 10^−6^	0.00068	3
CHST9 *	18q11.2	rs1436904	3.00 × 10^−8^	1.52 × 10^−11^	3
***CNTNAP1***	17q21.2	rs72826962	5.00 × 10^−9^	8.69 × 10^−9^	3
***DNAH11***	7p15.3	rs7971	2.00 × 10^−8^	3.32 × 10^−8^	3

* Indicates that the gene contains germline mutations that have been directly associated with TNBC.

## Data Availability

GWAS data is provided in SA provided as Table SA provided as Supplementary Materials to this report. Additional GWAS information is available at the GWAS catalog managed by the European Bioinformatics Institute: https://www.ebi.ac.uk/gwas/. Original gene expression and mutation data are available at the TCGA via the Genomics Data Commons. https://gdc.cancer.gov/.

## Supplementary Materials

The following are available online at https://www.mdpi.com/1660-4601/16/6/1055/s1, Figure S1: Patterns of gene expression profiles for the 366 genes. The heat map includes 267 genes from significantly differentially expressed genes containing only germline mutations and 99 somatic-mutated genes found to be highly significantly differentially expressed and have >2 somatic mutation events per gene, Figure S2: Patterns of gene expression for 237 genes containing both germline and somatic mutations, 267 genes containing germline mutations only, and 99 highly somatic-mutated genes significantly associated with TNBC. Table SA: List of germline mutations and genes associated with and increased of developing breast cancer reported in GWAS used in this study. The table included original published reports curated from the literature and from GWAS catalog database. Also presented in the table are estimates of GWAS *p*-values showing the strength of association and the information on original reports from which the genetic variants and the genes were derived, Table S1A: List of significantly differentially expressed somatic-mutated genes along with the frequency of somatic events, observed and adjusted *p*-values, Table S1B: List of significantly differentially expressed genes without mutations along with observed and adjusted *p*-values, Table S2: List of all 632 significantly differentially and not differentially expressed germline-mutated genes only. *p*-values of significantly differentially expressed genes between patients with TNBC and control samples are marked in red font. Also included in the table is the number of somatic mutation events per gene, Table S3: List of 237 significantly differentially expressed germline and somatic-mutated genes associated with TNBC using expression data, along with estimates of adjusted *p*-values and number of somatic events per gene, Table S4A: List of the 99 somatic-mutated genes significantly associated with TNBC using Figure 4 along with their estimates of *p*-values and number of somatic mutation events. Note that apart from the *p*-value all these genes have a fold change of ≥ 2.0 as indicated by the logFC, Table S4B: List of the 55 germline-mutated genes significantly associated with TNBC used in Figure 4 along with their estimates of *p*-values. Note that apart from the *p*-value all these genes have a fold change of ≥ 2.0 as indicated by the logFC.

## References

[1] Dietze E.C., Chavez T.A., Seewaldt V.L. (2018). Obesity and Triple-Negative Breast Cancer. Am. J. Pathol..

[2] Dietze E.C., Sistrunk C., Miranda-Carboni G., O’Regan R., Seewaldt V.L. (2015). Triple-negative breast cancer in African-American women: Disparities versus biology. Nat. Rev. Cancer.

[3] Perou C.M. (2010). Molecular Stratification of Triple-Negative Breast Cancers. Oncologist.

[4] Xu H., Eirew P., Mullaly S.C., Aparicio S. (2014). The omics of triple-negative breast cancers. Clin. Chem..

[5] Stevens K.N., Vachon C.M., Couch F.J. (2013). Genetic susceptibility to triple-negative breast cancer. Cancer Res..

[6] Lehmann B.D., Pietenpol J.A. (2014). Identification and use of biomarkers in treatment strategies for triple-negative breast cancer subtypes. J. Pathol..

[7] Lehmann B.D., Jovanović B., Chen X., Estrada M.V., Johnson K.N., Shyr Y., Moses H.L., Sanders M.E., Pietenpol J.A. (2016). Refinement of Triple-Negative Breast Cancer Molecular Subtypes: Implications for Neoadjuvant Chemotherapy Selection. PLoS ONE.

[8] Carter H., Marty R., Hofree M., Gross A.M., Jensen J., Fisch K.M., Wu X., DeBoever C., Van Nostrand E.L., Song Y. (2017). Interaction Landscape of Inherited Polymorphisms with Somatic Events in Cancer. Cancer Discov..

[9] Zhu B., Mukherjee A., Machiela M.J., Song L., Hua X., Shi J., Garcia-Closas M., Chanock S.J., Chatterjee N. (2016). An investigation of the association of genetic susceptibility risk with somatic mutation burden in breast cancer. Br. J. Cancer.

[10] Hicks C., Kumar R., Pannuti A., Backus K., Brown A., Monico J., Miele L. (2013). An Integrative Genomics Approach for Associating GWAs Information with Triple-negative Breast cancer. Cancer Inform..

[11] Purrington K.S., Slager S., Eccles D., Yannoukakos D., Fasching P.A., Miron P., Carpenter J., Chang-Claude J., Martin N.G., Montgomery G.W. (2014). Genome-wide association study identifies 25 known breast cancer susceptibility loci as risk factors for triple-negative breast cancer. Carcinogenesis.

[12] Shah S.P., Roth A., Goya R., Oloumi A., Ha G., Zhao Y., Turashvili G., Ding J., Tse K., Haffari G. (2012). The clonal and mutational evolution spectrum of primary triple-negative breast cancers. Nature.

[13] Weinstein J.N., Collisson E.A., Mills G.B., Shaw K.R.M., Ozenberger B.A., Ellrott K., Shmulevich I., Sander C., Stuart J.M., Cancer Genome Atlas Research Network (2013). The Cancer Genome Atlas Pan-Cancer analysis project. Nat. Genet..

[14] Hudson T.J., Anderson W., Aretz A., Barker A.D., Bell C., Bernabé R.R., Bhan M.K., Calvo F., Eerola I., Gerhard D.S. (2010). International network of cancer genome projects. Nature.

[15] The NHGRI-EBI Catalog of Published Genome-Wide Association Studies. https://www.ebi.ac.uk/gwas/.

[16] Ioannidis J.P., Boffetta P., Little J., O’Brien T.R., Uitterlinden A.G., Vineis P., Balding D.J., Chokkalingam A., Dolan S.M., Flanders W.D. (2008). Assessment of cumulative evidence on genetic associations: Interim guidelines. Int. J. Epidemiol..

[17] Khoury M.J., Bertram L., Boffetta P., Butterworth A.S., Chanock S.J., Dolan S.M., Fortier I., Garcia-Closas M., Gwinn M., Higgins J.P.T. (2009). Genome-Wide Association Studies, Field Synopses, and the Development of the Knowledge Base on Genetic Variation and Human Diseases. Am. J. Epidemiol..

[18] Sagoo G.S., Little J., Higgins J.P.T. (2009). Systematic Reviews of Genetic Association Studies. PLoS Med..

[19] Moher D., Liberati A., Tetzlaff J., Altman D.G., Group T.P. (2009). Preferred Reporting Items for Systematic Reviews and Meta-Analyses: The PRISMA Statement. PLoS Med..

[20] Liberati A., Altman D.G., Tetzlaff J., Mulrow C., Gøtzsche P.C., Ioannidis J.P.A., Clarke M., Devereaux P.J., Kleijnen J., Moher D. (2009). The PRISMA Statement for Reporting Systematic Reviews and Meta-Analyses of Studies That Evaluate Health Care Interventions: Explanation and Elaboration. PLoS Med..

[21] The Short Genetic Variations Database (dbSNP). https://www.ncbi.nlm.nih.gov/snp.

[22] Human Genome Nomenclature Committee (HGNC). https://www.genenames.org/.

[23] NCI Genomics Data Commons. https://gdc.cancer.gov/.

[24] Ritchie M.E., Phipson B., Wu D., Hu Y., Law C.W., Shi W., Smyth G.K. (2015). limma powers differential expression analyses for RNA-sequencing and microarray studies. Nucleic Acids Res..

[25] Benjamini Y., Hochberg Y. (1995). Controlling the False Discovery Rate : A Practical and Powerful Approach to Multiple Testing. J. R. Stat. Soc. Ser. B.

[26] Morpheus. https://software.broadinstitute.org/morpheus/.

[27] Ingenuity Pathways Analysis (IPA) System https://www.qiagenbioinformatics.com/products/ingenuity-pathway-analysis/.

[28] Ashburner M., Ball C.A., Blake J.A., Botstein D., Butler H., Cherry J.M., Davis A.P., Dolinski K., Dwight S.S., Eppig J.T. (2000). Gene ontology: Tool for the unification of biology. The Gene Ontology Consortium. Nat. Genet..

[29] Hahnen E., Lederer B., Hauke J., Loibl S., Kröber S., Schneeweiss A., Denkert C., Fasching P.A., Blohmer J.U., Jackisch C. (2017). Germline Mutation Status, Pathological Complete Response, and Disease-Free Survival in Triple-Negative Breast Cancer: Secondary Analysis of the GeparSixto Randomized Clinical Trial. JAMA Oncol..

[30] Petrucelli N., Daly M.B., Feldman G.L. (2010). Hereditary breast and ovarian cancer due to mutations in BRCA1 and BRCA2. Genet. Med..

[31] De Leeneer K., Coene I., Crombez B., Simkens J., Van den Broecke R., Bols A., Stragier B., Vanhoutte I., De Paepe A., Poppe B. (2012). Prevalence of BRCA1/2 mutations in sporadic breast/ovarian cancer patients and identification of a novel de novo BRCA1 mutation in a patient diagnosed with late onset breast and ovarian cancer: Implications for genetic testing. Breast Cancer Res. Treat..

[32] Engel C., Rhiem K., Hahnen E., Loibl S., Weber K.E., Seiler S., Zachariae S., Hauke J., Wappenschmidt B., Waha A. (2018). Prevalence of pathogenic BRCA1/2 germline mutations among 802 women with unilateral triple-negative breast cancer without family cancer history. BMC Cancer.

[33] Hauke J., Horvath J., Groß E., Gehrig A., Honisch E., Hackmann K., Schmidt G., Arnold N., Faust U., Sutter C. (2018). Gene panel testing of 5589 BRCA1/2-negative index patients with breast cancer in a routine diagnostic setting: Results of the German Consortium for Hereditary Breast and Ovarian Cancer. Cancer Med..

[34] Shimelis H., LaDuca H., Hu C., Hart S.N., Na J., Thomas A., Akinhanmi M., Moore R.M., Brauch H., Cox A. (2018). Triple-Negative Breast Cancer Risk Genes Identified by Multigene Hereditary Cancer Panel Testing. J. Natl. Cancer Inst..

[35] Mavaddat N., Michailidou K., Dennis J., Lush M., Fachal L., Lee A., Tyrer J.P., Chen T.-H., Wang Q., Bolla M.K. (2019). Polygenic Risk Scores for Prediction of Breast Cancer and Breast Cancer Subtypes. Am. J. Hum. Genet..

[36] Churbanov A., Vorechovský I., Hicks C. (2010). A method of predicting changes in human gene splicing induced by genetic variants in context of cis-acting elements. BMC Bioinform..

[37] Chen H., Li C., Peng X., Zhou Z., Weinstein J.N., Caesar-Johnson S.J., Demchok J.A., Felau I., Kasapi M., Ferguson M.L. (2018). A Pan-Cancer Analysis of Enhancer Expression in Nearly 9000 Patient Samples. Cell.

[38] Bonifaci N., Górski B., Masojć B., Wokołorczyk D., Jakubowska A., Dębniak T., Berenguer A., Serra Musach J., Brunet J., Dopazo J. (2010). Exploring the link between germline and somatic genetic alterations in breast carcinogenesis. PLoS ONE.

[39] Grünewald T.G.P., Delattre O. (2016). Cooperation between somatic mutations and germline susceptibility variants in tumorigenesis—A dangerous liaison. Mol. Cell. Oncol..

[40] Waszak S.M., Tiao G., Zhu B., Rausch T., Muyas F., Rodriguez-Martin B., Rabionet R., Yakneen S., Escaramis G., Li Y. (2017). Germline Determinants of the Somatic Mutation Landscape in 2642 Cancer Genomes.

[41] Wallden B., Storhoff J., Nielsen T., Dowidar N., Schaper C., Ferree S., Liu S., Leung S., Geiss G., Snider J. (2015). Development and verification of the PAM50-based Prosigna breast cancer gene signature assay. BMC Med. Genom..

[42] Liu M.C., Pitcher B.N., Mardis E.R., Davies S.R., Friedman P.N., Snider J.E., Vickery T.L., Reed J.P., DeSchryver K., Singh B. (2016). PAM50 gene signatures and breast cancer prognosis with adjuvant anthracycline- and taxane-based chemotherapy: Correlative analysis of C9741 (Alliance). NPJ Breast Cancer.

[43] Machiela M.J., Ho B.M., Fisher V.A., Hua X., Chanock S.J. (2015). Limited evidence that cancer susceptibility regions are preferential targets for somatic mutation. Genome Biol..

[44] Njiaju U.O., Olopade O.I. (2012). Genetic Determinants of Breast Cancer Risk: A Review of Current Literature and Issues Pertaining to Clinical Application. Breast J..

[45] Landry L.G., Ali N., Williams D.R., Rehm H.L., Bonham V.L. (2018). Lack Of Diversity In Genomic Databases Is A Barrier To Translating Precision Medicine Research Into Practice. Health Aff..

