# Supplementary material for: Integrating Germline and Somatic Mutation Information for the Discovery of Biomarkers in Triple-Negative Breast Cancer
Source: Int J Environ Res Public Health. 2019 Mar 23;16(6):1055. doi: 10.3390/ijerph16061055 (PMC6466377; doi:10.3390/ijerph16061055)
Supplement: Supplementary file 1 [file ijerph-16-01055-s001.zip › ijerph-463945 Supplementary Figures.pdf]

International Journal of Environmental Research and Public Health

Supplementary Materials

Integrating Germline and Somatic Mutation Information for the Discovery of Biomarkers in Triple Negative Breast Cancer

Jiande Wu [1], Tarun Karthik Kumar Mamidi [1], Lu Zhang [2] and Chindo Hicks [1,*]

International Journal of
Environmental Research and Public Health

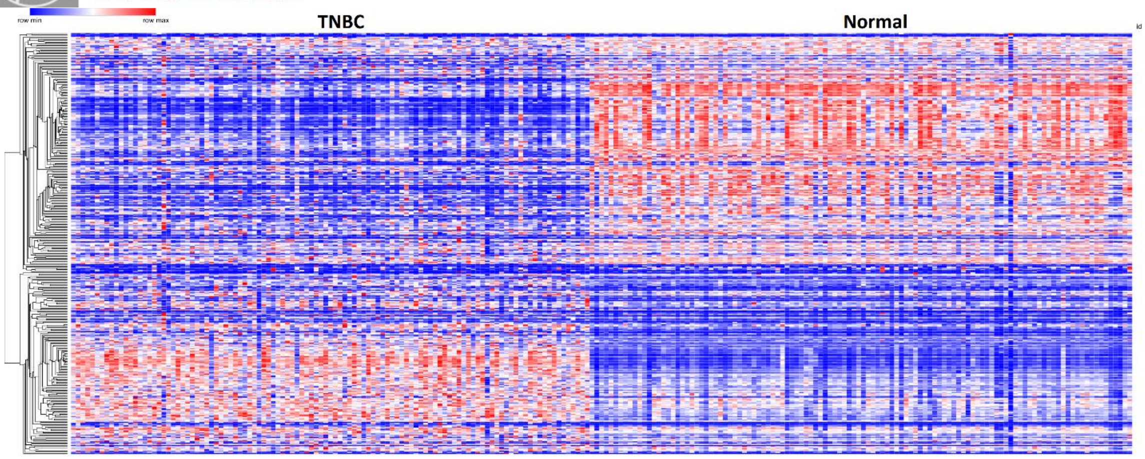

Figure S1. Patterns of gene expression profiles for the 366 genes. The heat map includes 267 genes from significantly differentially expressed genes containing only germline mutations and 99 somatic mutated genes found to be highly significantly differentially expressed and have > 2 somatic mutation events per gene.

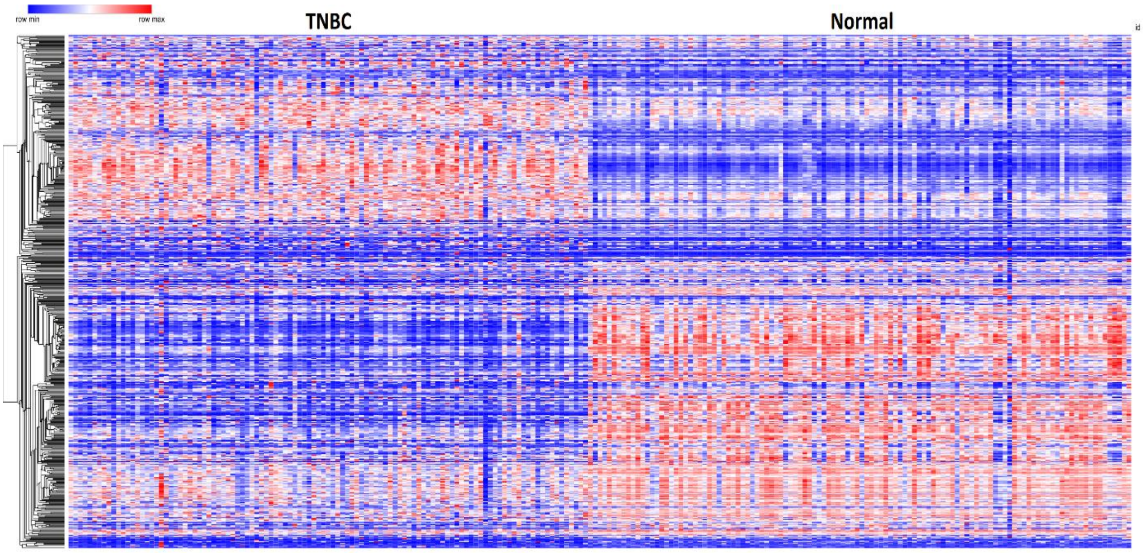

Figure 2. Patterns of gene expression for 237 genes containing both germline and somatic mutations, 267 genes containing germline mutations only and 99 highly somatic mutated genes significantly associated with TNBC.
